# TAILOR – tapered discontinuation versus maintenance therapy of antipsychotic medication in patients with newly diagnosed schizophrenia or persistent delusional disorder in remission of psychotic symptoms: study protocol for a randomized clinical trial

**DOI:** 10.1186/s13063-017-2172-4

**Published:** 2017-09-29

**Authors:** Anne Emilie Stürup, Heidi Dorthe Jensen, Signe Dolmer, Merete Birk, Nikolai Albert, Mai Nielsen, Carsten Hjorthøj, Lene Eplov, Bjørn H. Ebdrup, Ole Mors, Merete Nordentoft

**Affiliations:** 1 Copenhagen University Hospital, Mental Health Center Copenhagen, Kildegårsvej 28, opg. 15 4. sal, 2900 Hellerup, Denmark; 2 University of Copenhagen, Institute for Clinical Medicine, Faculty of Health and Medical Science, Blegdamsvej 3B, 2200 Copenhagen N, Denmark; 3 Psychosis Research Unit, Aarhus University Hospital, Skovagervej 2, 8240 Risskov, Denmark; 4 Centre for Clinical Intervention and Neuropsychiatric Schizophrenia Research (CINS) and Centre for Neuropsychiatric Schizophrenia Research (CNSR), Mental Health Centre Glostrup, Copenhagen University Hospital, Nordre Ringvej 69, 2600 Glostrup, Denmark

**Keywords:** Schizophrenia, First-episode psychosis, Early intervention, Antipsychotic medication, Discontinuation, Tapering, Maintenance therapy, Minimal effective dose, Randomized clinical trial

## Abstract

**Background:**

The aim of the TAILOR trial is to investigate the effect of closely monitored tapering/discontinuation versus maintenance therapy with antipsychotic medication in patients with newly diagnosed schizophrenia or persistent delusional disorder and with minimum 3 months’ remission of psychotic symptoms.

**Methods and design:**

Two hundred and fifty patients will be included from the psychiatric early intervention program, OPUS, in two regions in Denmark. Inclusion criteria are: ICD-10 diagnoses schizophrenia (F20, except F20.6) or persistent delusional disorder (F22), minimum 3 months’ remission of psychotic symptoms and in treatment with antipsychotic medication (except clozapine). The patients will be randomized to maintenance therapy or tapering/discontinuation with antipsychotic medication in a 1-year intervention. The tapering/discontinuation group will be using a smartphone application to monitor early warning signs of psychotic relapse. Patients will be assessed at baseline, 1-, 2- and 5-year follow-up regarding psychotic and negative symptoms, side-effects of antipsychotic medication, social functioning, cognitive functioning, perceived health status, patient satisfaction, substance and alcohol use, sexual functioning and quality of life. The primary outcome will be remission of psychotic symptoms and no antipsychotic medication after 1 year. Secondary outcome measures will include: co-occurrence of remission of psychotic symptoms and 0–1-mg haloperidol equivalents of antipsychotic medication after 1-year intervention; antipsychotic dose; antipsychotic side effects; negative symptoms; social functioning; cognitive functioning; and patient satisfaction. Exploratory outcomes will include remission, clinical recovery, substance and alcohol use, sexual functioning, quality of life, self-beliefs of coping and user experience of support from health workers. Safety measures will include death, admissions to psychiatric hospital, severe self-harm and psychotic relapses.

**Discussion:**

The TAILOR trial will contribute knowledge about the effect of tapering/discontinuation of antipsychotic medication in the early phases of schizophrenia and related disorders and the results may guide future clinical treatment regimens of antipsychotic treatment.

**Trial registration:**

EU Clinical Trials Register – EudraCT number: 2016-000565-23. Registered on 5 February 2016.

**Electronic supplementary material:**

The online version of this article (doi:10.1186/s13063-017-2172-4) contains supplementary material, which is available to authorized users.

## Background

Schizophrenia spectrum disorders have major implications for the individual, family and society [1]. The clinical features include psychotic symptoms (hallucinations and delusions), negative symptoms (e.g., alogia, affective flattening, and avolition) and cognitive impairment of, e.g., memory and social cognition. All symptom domains may exert a severe impact on level of functioning and quality of life [2, 3].

Antipsychotic medication is often effective in the treatment of psychotic symptoms in schizophrenia spectrum disorders [4], but it has questionable effects on negative symptoms [5] and limited effect on cognitive function [6, 7]. Antipsychotics often reduce the risk of relapse after remission of psychotic symptoms – at least in the short term [8]. This is the rationale for recommending maintenance treatment with antipsychotic medication in national and international guidelines for the treatment of schizophrenia [4, 9, 10]. However, current guidelines do not provide recommendations as to which patients might be able to discontinue their antipsychotic medication without relapsing. More knowledge is needed to give specific and tailored recommendations to the individual about treatment with antipsychotic medication.

A large meta-analysis of randomized controlled trials investigated the effect of antipsychotic medication versus placebo on relapse prevention [8]. The authors found that antipsychotic medication reduced the risk of relapse with 60% in patients with a long duration of schizophrenia and with 47% in patients with first-episode schizophrenia. The meta-analysis indicated that the difference of relapse in treatment with antipsychotic medication versus placebo was lower in studies lasting more than 2 years [8]. The risk of relapse was not influenced by the duration of time in stable remission before study entrance, or whether the patient was in remission or not. Similar results were later published in a meta-analysis solely on non-affective first-episode psychosis [11].

There are, however, risks of side effects of all antipsychotic medication such as movement disorders, weight gain, diabetes, cardiovascular disease, sedation and decreased libido [8, 12, 13]. The degree of side effects depends on drug, and sometimes, dose [14]. Many side effects are reversible but some movement disorders, such as tardive dyskinesia or tardive dystonia, may be irreversible. Obesity, hyperlipidaemia and diabetes are often persistent side effects and it is unclear whether they are irreversible. Especially, second-generation antipsychotics, which are first-line treatment in schizophrenia, cause metabolic side effects and a higher risk of cardiovascular morbidity [14, 15]. This is especially unwanted because patients with schizophrenia already have an increased morbidity and mortality [16–18]. The side effects of antipsychotic medication are generally associated with reduced quality of life [19].

Studies of first-episode psychosis have shown that some 50 to 60% of the patients were in remission regarding psychotic symptoms at 10-year follow-up [20, 21] and approximately half of the patients had discontinued antipsychotic medication [21, 22]. The studies showed that a substantial group of patients with first-episode psychosis in the long term can manage without antipsychotic medication.

A randomized trial of 128 patients compared maintenance treatment with antipsychotic medication to dose reduction [23]. At 7-year follow-up more than 40% of patients in the dose-reduction group were in stable remission with no or maximum 1-mg haloperidol equivalent antipsychotic medication daily compared to only 20% in the maintenance group [24]. The trial revealed that the dose-reduction group had a higher risk of relapse in the beginning of the trial but the relapses did not cause long hospital admissions and the difference in relapse rate in the two groups was non-significant at 7-year follow-up [23–26].

In the TAILOR trial we want to compare maintenance treatment with closely monitored tapering/discontinuation of antipsychotic medication in patients with schizophrenia or persistent delusional disorder. These disorders were chosen because they both have prolonged durations of psychotic experiences and the treatment recommendations for the disorders are similar. Thereby, we will evaluate whether it is possible to reduce dose and maybe discontinue without relapse of psychotic symptoms. Furthermore, the TAILOR trial will describe the effects of tapering on negative symptoms, dose of antipsychotic medication, side effects of antipsychotic medication, social functioning, recovery, cognitive functioning, patient satisfaction, substance and alcohol use, sexual functioning, quality of life, self-belief of coping, patients’ experience of support from health workers, relapses, death and hospital admissions. We hope that the TAILOR trial contributes to knowledge about which patients can manage with little or no antipsychotic medication.

## Methods and design

**Fig. 1:**
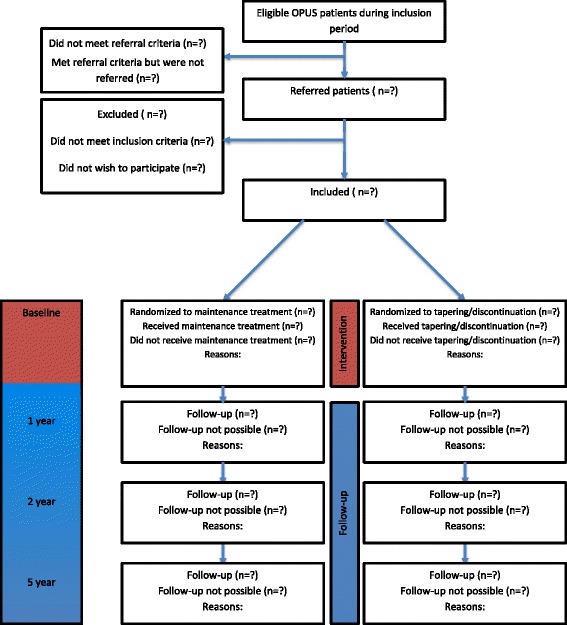
Flow chart of the TAILOR trial

This paper was written in line with the SPIRIT (Standard Protocol Items: Recommendations for Interventional Trials) 2013 explanation and elaboration: guidance for protocols of clinical trials [27] and the SPIRIT Checklist and flow chart were used, see Additional file 1 and Fig. 1.

### Aim and objectives

The aim of the TAILOR trial is to investigate the effect of closely monitored tapering/discontinuation versus maintenance therapy with antipsychotic medication in patients with newly diagnosed schizophrenia or persistent delusional disorder and with minimum 3 months’ remission of psychotic symptoms.

In the TAILOR trial the following hypotheses will be tested:

- Primary hypothesis:
  1. After 1 year there will be more patients in the tapering/discontinuation group than in the maintenance group who will not take antipsychotic medication and will be in remission of psychotic symptoms (null hypothesis: no difference in the tapering/discontinuation group compared to the maintenance group regarding number of patients not taking antipsychotic medication with remission of psychotic symptoms at 1-year follow-up)
- Secondary hypotheses:
  1. After 2 years there will be more patients in the tapering/discontinuation group than in the maintenance group who will not take antipsychotic medication and will be in remission of psychotic symptoms (null hypothesis: no difference in the tapering/discontinuation group compared to the maintenance group regarding number of patients not taking antipsychotic medication with remission of psychotic symptoms at 2-year follow-up)
  2. After the first, second and fifth years there will be more psychotic relapses in the tapering/discontinuation group than in the maintenance group (null hypothesis: no difference in the tapering/discontinuation group compared to the maintenance group regarding number of patients with a psychotic relapse after 1, 2 and 5 years)
  3. The prevalence of side effects of antipsychotic medication at 1-, 2- and 5-year follow-up will be lower in the tapering/discontinuation group than in the maintenance group (null hypothesis: no difference in the tapering/discontinuation group compared to the maintenance group regarding side effects after 1, 2 and 5 years)
  4. Null hypothesis: level of social functioning will be the same in the tapering/discontinuation group and in the maintenance group after 1, 2 and 5 years
  5. Null hypothesis: cognitive functioning will be the same in the tapering/discontinuation group and in the maintenance group after 1, 2 and 5 years
  6. Null hypothesis: clinical recovery will be the same in the tapering/discontinuation group and in the maintenance group after 2 and 5 years
  7. Null hypothesis: substance and alcohol use will be the same in the tapering/discontinuation group and in the maintenance group after 1, 2 and 5 years

### Trial design and setting

The TAILOR trial is an investigator-initiated, randomized, multicentre, assessor-blinded, parallel-group, superiority designed clinical trial. The trial subjects, their physicians and contact persons in the OPUS teams will not be blinded to which treatment the trial subjects are randomized to, but the researchers will be blinded [28].

The trial subjects will receive 1-year intervention in their psychiatric outpatient treatment team, OPUS, in the Capital Region of Denmark and The Central Denmark Region. A list of trial sites can be obtained at the sponsor. OPUS is an outpatient treatment modality for people with first-episode psychosis, e.g., schizophrenia. It comprises treatment with antipsychotic medication, cognitive-based case management, psychoeducation, family involvement, social skills training and integration in society [29].

The researchers will be physicians and other health professionals, trained in the instruments for the assessments at inclusion and follow-ups. The health professionals performing the intervention will work at the OPUS team and the psychiatrist at the OPUS team will have the treatment responsibility for the patient.

### Study population and eligibility criteria

- Inclusion criteria:
  1. First treatment in an OPUS team with the *International Classification of Diseases, version 10* (ICD-10) diagnosis schizophrenia (F20, except F20.6) or persistent delusional disorder (F22). The diagnosis will be established by the researcher with the diagnostic semistructured interview SCAN (Schedule for Clinical Assessment I Neuropsychiatry [30])
  2. Minimum 3 months’ remission of psychotic symptoms and within the first 11 months of treatment in the OPUS team. The researchers will confirm remission with SAPS (Schedule for Assessment of Positive Symptoms in Schizophrenia [31]) (all global scores below 3)
  3. In treatment with antipsychotic medicine (daily or depot)
  4. Minimum age 18 years, fluent in Danish and informed consent
- Exclusion criteria:
  1. Patients in forensic psychiatry, because Danish law prohibits any interference with treatment
  2. Using treatment with clozapine, which indicates treatment resistance, why tapering/discontinuation will carry a high risk of psychotic relapse
  3. Pregnancy or breastfeeding
  4. Previous admission to a psychiatric hospital due to a psychotic relapse while treated with antipsychotic medication or tapering of antipsychotic medication
- Criteria for discontinuing the intervention:
  If the patient, within the year of intervention, meets exclusion criteria 1, 2 or 3, is no longer treated in an OPUS team or withdraws informed consent, they will be excluded from the intervention. The patient will still be assessed at follow-up or, in case of withdrawal of consent, be asked to participate in the follow-up interviews.

### Interventions

During the 1-year intervention we compare antipsychotic maintenance treatment with tapering/discontinuation of antipsychotic medication. The inclusion process is illustrated in Fig. 1. Both intervention groups will be in continuous contact with their OPUS team during the intervention year and receive usual non-pharmacological treatment in their OPUS team and one of (or tapering from one of) the following antipsychotics (active substance): amisulpride, aripiprazole, chlorprothixene, haloperidol, haloperidol decanoate, lurasidone, olanzapine, paliperidone, paliperidone palmitate, perphenazine, perphenazine decanoate, quetiapine, risperidone, sertindole, ziprasidone, zuclopenthixol or zuclopenthixol decanoate.

Maintenance treatment:

In Denmark, maintenance treatment is recommended for at least 1 year after remission [4, 9, 10] and patients in the maintenance group, therefore, receive treatment as usual. In the intervention year the patients will continue taking antipsychotic medication with the possibility to switch to another antipsychotic medication (the same dose in haloperidol equivalents) and change dose.

If the effect or side effects of antipsychotic medication require discontinuation, it is allowed to modify intervention, and if so, the patient stays in the maintenance group.

**Fig. 2:**
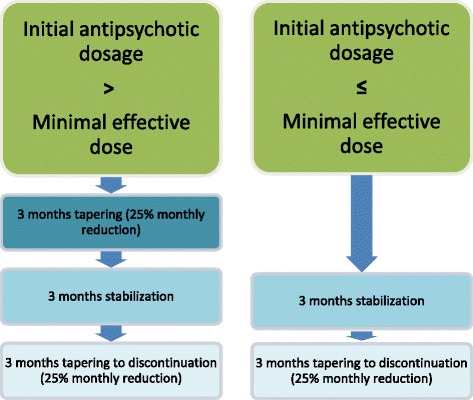
The tapering/discontinuation intervention

Tapering/discontinuation:

Tapering and eventual discontinuation of antipsychotic medication is managed by the treatment responsible psychiatrist in the OPUS team. The tapering is tailored to be: (1) approximately 25% monthly reduction of initial dose, (2) with at least five half lives between each reduction, (3) duration of minimum 6 months and (4) with regular assessments and evaluations. If the initial dose is above the Minimum Effective Dose (MED) [32] the first step is 3 months’ tapering to MED, next step dose is maintained in a 3-month stabilization phase, before the final step of 3 months’ tapering to discontinuation. If the initial dose is below MED the first step is skipped. The tapering/discontinuation is illustrated in Fig. 2.

The patients will be receive a user-developed mobile phone application to make daily registrations. A next of kin can add a daily note which will be visible to the patient, and the health professional at the OPUS team can access all data from a web portal. The aim is to discover early warning signs of relapse and thereby enhancing safety.

If the patients experience deterioration (increase in SAPS from 0/1 to 2 or above, or from 2 to 3 or above, or individual warning signs evaluated by the clinician as signs of deterioration) or relapse (see definition in the “Outcomes” section below) it is recommended to increase the dose to the most recent effective dose or, if necessary, above this. Tapering can be resumed after 3 months of remission of psychotic symptoms; however, this is only if the patient and physician agree on this. Despite deterioration or relapse the patient stays in the tapering/discontinuation group. If a switch of antipsychotic medication is necessary it is recommended that the dose of the new antipsychotic is the same in haloperidol equivalents as the previous antipsychotic.

To improve adherence patients will have regular visits to their OPUS team. To control for this the patients’ blood level of antipsychotics will be checked at baseline and follow-up. The researchers will do a SAPS phone interview monthly to evaluate remission of psychotic symptoms.

### Outcomes

- Primary outcome:
  Remission of psychotic symptoms (SAPS ≤ 2 in all global scores in minimum 3 months) and no antipsychotic medication in 3 months, assessed at 1-year follow-up.
- Secondary outcomes:
  1. Remission of psychotic symptoms (SAPS ≤ 2 in all global scores in minimum 3 months) and antipsychotic medication > 0- and ≤ 1-mg haloperidol equivalents any given day in the 3 months before 1-year follow-up
  2. Antipsychotic dose (haloperidol equivalents) at 1-year follow-up
  3. Antipsychotic side effects at 1-year follow-up
  4. Negative symptoms measured with the SANS (Schedule for Assessment of Negative Symptoms [33]) at 1-year follow-up
  5. Social functioning measured with the GSDS (Groningen Social Disabilities Schedule [34]) at 1-year follow-up
  6. Cognitive functioning measured with the BACS (Brief Assessment of Cognition in Schizophrenia [35]) at 1-year follow-up
  7. Patient satisfaction measured with the CSQ (Patient Satisfaction Questionnaire [36]) at 1-year follow-up
- Explorative outcomes:
  1. Remission of psychotic symptoms (SAPS ≤ 2 in all global scores in a minimum of 3 months) and no antipsychotic medication in 3 months, assessed at 2- and 5-year follow-up
  2. Remission of psychotic symptoms (SAPS ≤ 2 in all global scores in a minimum of 3 months) and antipsychotic medication > 0- and ≤ 1-mg haloperidol equivalents on any given day in the 3 months before 2- and 5-year follow-up
  3. Antipsychotic dose (haloperidol equivalents) at 2- and 5-year follow-up
  4. Antipsychotic side effects at 2- and 5-year follow-up
  5. Negative symptoms measured with the SANS at 2- and 5-year follow-up
  6. Social functioning measured with the GSDS at 2- and 5-year follow-up
  7. Social functioning measured with the PSP (Personal and Social Performance Scale [37]) and the GAF (Global Assessment of Function [38]) at 1-, 2- and 5-year follow-up
  8. Cognitive functioning measured with the BACS at 2- and 5-year follow-up
  9. Remission measured at 1-, 2- and 5-year follow-up: remission of psychotic symptoms (SAPS ≤ 2 in all global scores in a minimum of 3 months), negative symptoms (SANS ≤ 2 in all global scores in a minimum of 3 months) and functional remission [39] (GSDS ≤ 1 in all roles simultaneously in a minimum of 3 months)
  10. Remission measured at 1-, 2- and 5-year follow-up: remission of psychotic and negative symptoms in a minimum of 6 months and functional remission in a minimum of 6 months
  11. Clinical recovery [39] at 2-year follow-up: 2-year remission of both psychotic and negative symptoms and functional remission through 2 years and no admissions to psychiatric hospital the past 2 years [39]
  12. Substance and alcohol use measured with time line follow back [40] at 1-, 2- and 5-year follow-up
  13. Sexual functioning measured with the CSFQ (Changes in Sexual Functioning Questionnaire [41]) and the SUSY questionnaire from the Danish Health Interview Survey at 1-, 2- and 5-year follow-up
  14. Quality of life measured with the WHO-5 Well-being Index (WHO-5) [42] and a question about self-rated health at 1-, 2- and 5-year follow-up
  15. Self-belief of coping measured with the GSE (General Self Efficacy [43]) at 1-, 2- and 5-year follow-up
  16. Patients’ experience of support from health workers measured with INSPIRE [44] at 1-year follow-up
  17. The EuroQol EQ-5D [45, 46] as a measure of health-related quality of life at 1, 2 and 5-year follow-up
- Safety measures:
  Incidents of death, suicide, admission to psychiatric hospital, persistent or significant disability/incapacity, severe self-harm and psychotic relapse (defined as all of the four following criteria fulfilled simultaneously: (1) clinical deterioration in a minimum of 1 week, (2) consequences (e.g., psychiatric hospital admission or more visits in the OPUS team), (3) clinician evaluates the episode as a relapse and (4) SAPS ≥ 4).
  One-year data from safety measures and primary, secondary and exploratory outcomes will all be analyzed and published. Thereafter, data from 2-year and, finally, 5-year follow-up will be analyzed and published.

### Sample size

We have conducted sample size calculations on the primary outcome measure and several of the secondary outcome measures. In all cases, we have applied a two-sided level of significance of 5%.

In the 1-year OPUS follow-up study, 73 patients received OPUS treatment, received antipsychotic medication, and were in remission of psychotic symptoms (SAPS ≤ 2) [22]. At the 2-year follow-up, 17 (23.3%) of these patients were still in remission but no longer took antipsychotic medication (See Table 1). This was achieved without a systematic effort towards dose reduction. This number of patients in this group is comparable to the expected number for our control group (see Table 1). Based on this number, the sample size calculation was performed with alpha = 0.05 and 80% power to detect the minimum relevant difference if the tapering/discontinuation group at 1-year follow-up has an event rate of 39.7% on remission without antipsychotic medication (see Table 1). This yields a required sample size of 125 patients in each group, i.e., a total of 250 patients in the TAILOR trial.

**Table 1:** Detectable differences for dichotomous outcome measures

Outcome	Tapering/discontinuation	Maintenance treatment	Reference
Remission and no antipsychotics (primary outcome)	39.7%	23.3%	Godtfredsen [21]
Stable remission of psychotic symptoms and max 1-mg haloperidol equivalents (most important secondary outcome measure)	15.4%	4.8%	Wunderink [22]
Percentage in recovery after 7 years (exploratory outcome measure)	33.4%	17.6%	Wunderink [23]

In a Dutch dose-reduction trial 21.5% of patients in the dose-reduction group were off medication (max 1-mg haloperidol equivalents daily) and in remission at 18-month follow-up, which was only the case for 4.8% in the maintenance-treatment group [23] (see Table 1). The 7-year follow-up of the same study showed that 17.6% of participants in the maintenance group achieved recovery, compared to 40.4% in the dose-reduction group [24]. For secondary outcome 1 (remission of psychotic symptoms and max 1 mg haloperidol daily) and exploratory outcome 11 (recovery), the power calculations in Table 1 are based on the references’ numbers for maintenance treatment, and the numbers for the tapering/discontinuation group is the minimally detectable difference with 80% power and 2 × 125 participants. For the continuous outcome measures we have estimated the power we have to detect the lowest clinically relevant difference indentified from previous studies at alpha = 0.05 to be 2 × 125 participants and the expected standard deviation from these studies (Table 2). Regarding our exploratory outcome on the GAF scale, we expect a standard deviation of 15 [47] and lowest clinically relevant difference of 5, for which we will have a power of 75%.

**Table 2:** Power calculations for continuous outcome measures

Outcome	Lowest clinically relevant difference	Expected standard deviation	Calculated power	Reference
Antipsychotic haloperidol equivalents	1.5	3	98%	Own unpublished data
Negative symptoms (negative dimension, based on mean of four global SANS scores)	0.4	1.2	75%	Petersen [45]
Cognition, overall score (BACS total)	15	42	80%	Melau [46]
Client satisfaction (CSQ)	2	5	88%	Petersen [45]
Social function (GSDS)	1.6	4.5	80%	Standard deviation based on [22]. Lowest clinically relevant difference is not known, but is set to Cohen’s *d* = 0.356, i.e.,low-to-moderate effect size

*CSQ* Patient Satisfaction Questionnaire, *BACS* Brief Assessment of Cognition in Schizophrenia, *GSDS*, Groningen Social Disabilities Schedule, *SANS* Schedule for Assessment of Negative Symptoms

### Recruitment and allocation

Participants will be recruited from OPUS teams and referred by team physicians (Fig. 1). To achieve a sufficient number of participants the physicians are informed about the TAILOR trial, distribute information material to the patients, and the TAILOR trial has a website with information. If necessary, the time for recruitment will be prolonged.

The randomization will take place after baseline interview and is centralized and computer-based with a hidden allocation sequence. The researchers and clinicians are blinded to the randomization block sizes. Factors for stratification are region (Capital Region of Denmark/The Central Denmark Region) and harmful use or dependence on psychoactive substances (ICD-10 diagnosis of F1x.1 and F1x.2 except F17.x) (yes/no). The program OPEN Randomize [48] will generate the randomization and use the correct randomization table from the four strata. Each patient enrolled in the TAILOR trial will be assigned a serial number before randomization and an email of assigned randomization will be sent to the health professionals performing the intervention in the OPUS team. A list of patients’ assigned randomization will be kept at the research team’s secretary. The randomization code will be stored at OPEN, Odense Patient data Explorative Network, at The University of Southern Denmark.

### Blinding

The researchers are blinded when performing assessments at baseline, during the intervention year and at follow-ups. If they are unblinded another researcher will do the assessment. The clinicians and patients are not blinded. The researchers will perform the analyses and draft of conclusions blinded. Unblinding is permissible and possibly necessary if serious adverse events (SAEs)), serious adverse reactions (SARs) or suspected unexpected serious adverse reactions (SUSARs) occur.

### Data collection methods and management

**Fig. 3:**
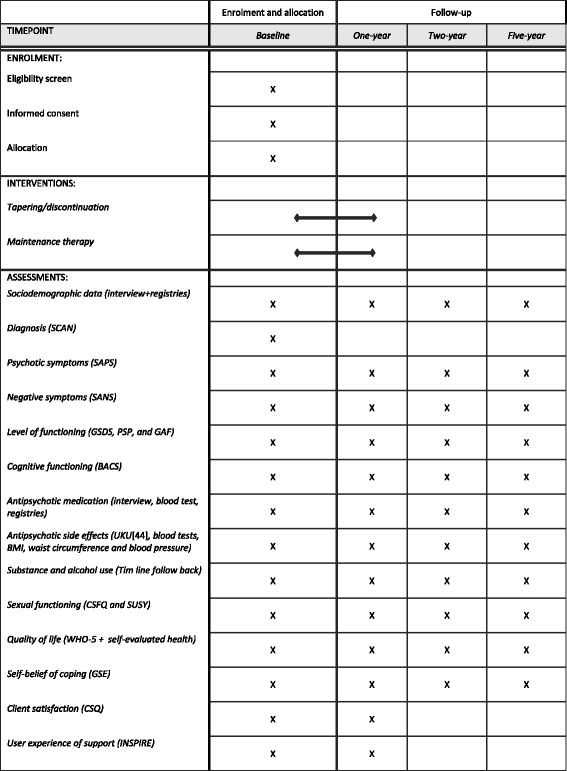
Standard Protocol Items: Recommendations for Interventional Trials (SPIRIT) Figure

Plans for collection of data at baseline and follow-up assessments are outlined in Fig. 3. Data on patients who do not complete the intervention will also be collected. Data Collection Forms are electronic in REDCap (Research Electronic Data Capture) [49] or on paper and stored by the principal investigator. To ensure data quality assessors are trained in the interview instruments, certified in the SCAN, the BACS and the GSDS and will do regular co-ratings of live interviews. Interrater reliability of the SAPS and the SANS will be calculated before the beginning of the TAILOR trial.

The assessors will enter data from the patient interview directly into the electronic Case Report Form (CRF) using the data entry system REDCap [49]. REDCap is an electronic data capture tool hosted at CIMT in the Capital Region of Denmark. When necessary, the collection will be done on paper and later entered electronically and with double data entry. REDCap has a complete audit trail on all data transactions, detailed user rights and access control management and thereby complies with Danish legislation (the Act on Processing Personal Data). Data for each patient is connected to a unique serial number. Only assigned researchers and Good Clinical Practice (GCP) monitors can access REDCap with all the data. Data on paper is stored locally and secured. Research data will be exported from REDCap without personal identifiers, assembled in a SPSS file and put in logged folders on a network drive under the control of the Capital Region of Denmark, CIMT. Sources of data are, for example, patient interview, blood sample values, waist circumference, blood pressure, patient files and registries. Sponsor and the principal investigators ensures that data is stored for at least 10 years after end of trial.

The patients participating in the TAILOR trial will receive a gift voucher at each follow-up, irrespective of randomization group, remission and medicine. This is to ensure participant retention and complete follow-up. The patients receive an invitation for follow-up in a letter, by email, or by a phone call.

### Statistical methods

The primary outcome measure will be analyzed with binary logistic regression to estimate the odds ratio for a successful tapering/discontinuation between the two groups. Other dichotomous outcome measures will likewise be analyzed with logistic regression. Continuous outcome measures will be controlled for whether they are normal distributed, and if so the comparison between groups will be done with general linear models. Non-parametric tests will performed on data which is not normally distributed. In all cases the following variables will be included in the analytical models: randomization (tapering/discontinuation versus maintenance), region (Central Denmark Region versus Capital Region of Denmark) and harmful use or dependence of psychoactive substances (yes versus no). The analyses will also include variables which, despite randomization, are skewed between the two groups. Interim analyses will not be done. All analyses will be by intention-to-treat and all included patients will be a part of the analyses regardless of whether they start the intervention or complete it. Missing data will be dealt with by multiple imputations. These imputations will be done with the baseline value of the variable and baseline values of variables which are predictive for attrition. Region (Central Denmark Region versus Capital Region of Denmark) and harmful use or dependence of psychoactive substances (yes versus no) will be part of the imputation models. Imputations will be done separately for each group. The continuous variables will be imputed with predictive mean matching with three nearest neighbors. For binary variables we will impute with binary logistic regression. For multinomial variables we will impute with multinomial logistic regression. For ordinal variables we will impute with ordinal logistic regression. In every case we will do 100 imputations. Analyses will be done after the last 1-year follow-up interview and will initially be done blinded for randomization and the groups will instead be named “A” and “B.”

The current publication of the original statistical procedures will ensure that the TAILOR trial is conducted and analyzed as planned. Possible deviations and reasons for those will be described in publications. All data published will be verified for authenticity by controlling internal inconsistency. All results, positive, negative as well as inconclusive, will be published as quick as possible and still in concordance with Danish law on protection of confidentially and personal information. Results will be presented at national and international scientific conferences.

### Data monitoring and auditing

The TAILOR trial is monitored by the GCP Unit at Aalborg and Aarhus University Hospitals and the GCP unit at Copenhagen University Hospital to ensure safety and data quality according to Good Clinical Practice – The International Council for Harmonization of Technical Requirements for Pharmaceuticals for Human Use (GCP-ICH) and Danish laws and regulations. The sponsor is responsible for the completion and quality of the TAILOR trial. The GCP units are independent of sponsor and investigators. The following describes the GCP units’ monitoring and auditing plan. The following data for included patients will be checked if they are correct: informed consent, inclusion, exclusion, randomization, allocation, exclusion from intervention, baseline at 1-year follow-up and the primary outcome measure. At 2- and 5-year follow-up a sampling of 10% of all included patients will be done regarding check of follow-up is done at the correct time. Every year it is checked that all relevant documents are in the trial master file. It will be monitored that assessments, analyses and procedures are done as described in the study protocol. It is also checked that all patients after a half year of intervention in the discontinuation/tapering group have reached MED or below and in the maintenance group still receive antipsychotic medication. All reports of SAEs or SARs are checked for completeness regarding time and information and that they have been evaluated by authorized personnel whether there is a link between trial medication and the SAEs or SARs. The principal investigators allow the GCP units, the Danish Medicines Agency and the Danish Health Authority access to data for monitoring, auditing and inspection. The plan for monitoring will be evaluated continuously if necessary.

### Safety

In the OPUS team the clinicians will, during the year of intervention, register adverse events (AEs)/adverse reaction (ARs) and report all SAEs/SARs to the regional investigator. It is the physician responsible for the antipsychotic treatment who will evaluate whether the AE/AR is considered to be a SAE/SAR. Other events or side effects will be collected from patient files and registers. SAEs, SARs and SUSARs are defined by the GCP-ICH guideline. Admission to a psychiatric hospital because of psychosis-related symptoms is to be expected in the TAILOR trial and will, although they are SAEs/SARs, only be reported yearly to the Danish Medical Agency and the Research Ethics Committee. The following will not be reported as SAEs/SARs: (1) clinical deterioration that does not result in admission to a psychiatric hospital, (2) ordinary side effects such as gastrointestinal side effects or headaches and (3) well-known discontinuation withdrawal effects such as weight loss, sleep disorders, fatigue, increased sweating, inattention, raised pulse or anxiety. In the TAILOR trial the reference to evaluate all events and reactions reported is the Summary of Product Characteristics of the antipsychotics. The evaluation of causality will be done by the physician responsible for treatment.

Four times a year all investigators are informed of all AEs/ARs in the trial. The sponsor will be informed of SEs/SARs within 24 h without regards to whether the event is related to the antipsychotic medication. The sponsor evaluates whether the SAE/SAR is a SUSAR. Every year during the trial Research Ethics Committee will receive a report of all SAEs/SARs and SUSARs and about the safety of the TAILOR trial. In case of a SUSAR the Research Ethics Committee will be informed immediately. The sponsor will inform the Danish Medicines Agency in case of a SUSAR and the reporting will be within 7 days in case of death or life-threatening condition or else within 15 days. All relevant information on the sponsor’s and investigators’ follow-up on the SUSAR will be reported to the Danish Medicines Agency no later than 8 days after the initial reporting. SAEs/SARs and SUSARs will be followed until they are dealt with. A committee consisting of the sponsor, principal investigators and representatives from outside the TAILOR trial will evaluate whether an SAE/SAR should result in end of trial. After the end of the trial the sponsor will make a report to the Danish Medicines Agency of all SAEs/SARs during the trial and about the patients’ safety. The investigator and sponsor will report all side effects and events to the Research Ethics Committee after the end of the trial.

The patients in the TAILOR trial are ensured by Danish law and the patient care regulation.

Every patient in the TAILOR trial will have access to their own results of the trial if they wish to. The clinicians will not have access to data collected from assessments done by the researchers.

## Discussion

The TAILOR trial raises ethical, practical and organizational challenges.

When designing the TAILOR trial ethical questions were raised regarding blinding and the design of the interventions. In the TAILOR trial only the researchers are blinded, neither clinicians nor patients, because they should be attentive of the high risk of relapse in the discontinuation group. The design of both interventions gives the clinicians the possibility to adjust the dose of the antipsychotic medication to ensure sufficient treatment. Therefore, the TAILOR trial only includes assessor blinding and the interventions might end up being more similar than intended and there is a risk that the patient and physician will misinterpret warnings signs or side effects. In general, it is of ethical consideration that the trial participants in the tapering/discontinuation group will be subjected to a higher risk of relapse. On the other hand, it seems unethical if research were not to discover the group of patients who can discontinue antipsychotic medication without relapsing. The trial participants in the maintenance group might be subjected to an intervention which no longer exists in the clinical setting and thereby be maintained on antipsychotic treatment longer than they would outside the experiment.

Practical challenges will be sufficient recruitment or patient dropout. Organizational structures will influence the completion of interventions as they are done in a clinical setting, which might influence recruitment and intervention.

The TAILOR trial is a complex medical intervention, which makes it difficult to know which components are more effective than others. We have ensured good fidelity by monitoring by the GCP Units in Aarhus and Copenhagen, but beyond this we have not planned a qualitative process evaluation.

Overall, we believe that the TAILOR trial will contribute to knowledge about the effect of tapering/discontinuation of antipsychotic medication in early phases of schizophrenia spectrum disorders and that the results may guide future clinical treatment regimens of antipsychotic medication.

## Trial status

Inclusion of patients will start in May 2017.

## Additional files

Additional file 1: SPIRIT Checklist. (DOCX 60 kb)

Additional file 2: Model Consent Form for Region Midt. (DOCX 33 kb)

Additional file 3: Model Consent Form for Region Hovedstaden. (DOCX 37 kb)

## Abbreviations

AE: Adverse event
AR: Adverse reaction
BACS: Brief Assessment of Cognition in Schizophrenia
CRF: Case Report Form
CSFQ: Changes in Sexual Functioning Questionnaire
CSQ: Patient Satisfaction Questionnaire
GAF: Global Assessment of Function
GCP: Good Clinical Practice
GCP-ICH: Good Clinical Practice - The International Council for Harmonization of Technical Requirements for Pharmaceuticals for Human Use
GSDS: Groningen Social Disabilities Schedule
GSE: General Self Efficacy
MED: Minimum effective dose
PSP: Personal and Social Performance Scale
SAE: Serious adverse event
SANS: Schedule for Assessment of Negative Symptoms
SAPS: Schedule for Assessment of Positive Symptoms
SAR: Serious adverse reaction
SCAN: Schedule for Clinical Assessment in Neuropsychiatry
SD: Standard deviation
SUSAR: Suspected unexpected serious adverse reaction
UKU: Udvalg for Kliniske Undersøgelser
