# Supplementary material for: TAILOR – tapered discontinuation versus maintenance therapy of antipsychotic medication in patients with newly diagnosed schizophrenia or persistent delusional disorder in remission of psychotic symptoms: study protocol for a randomized clinical trial
Source: Trials. 2017 Sep 29;18:445. doi: 10.1186/s13063-017-2172-4 (PMC5622425; doi:10.1186/s13063-017-2172-4)
Supplement: Supplementary file 2 — Model Consent Form for Region Midt. (DOCX 33 kb) [file 13063_2017_2172_MOESM2_ESM.docx]

**Informeret samtykke til deltagelse i et sundhedsvidenskabeligt forsøg**

Forsøgets titel: TAILOR – et randomiseret klinisk forsøg: Udtrapning versus vedligeholdelsesbehandling med antipsykotisk medicin hos patienter med ny-diagnosticeret skizofreni og skizofrenilignende psykose med remission af psykotiske symptomer

**Erklæring fra forsøgspersonen:**

Jeg har fået skriftlig og mundtlig information, og jeg ved nok om formål, metode, fordel og ulemper til at sige ja til at deltage.

Jeg ved, at det er frivilligt at deltage, og at jeg altid kan trække mit samtykke tilbage uden at miste mine nuværende eller fremtidige rettigheder til behandling.

Jeg giver samtykke til at deltage i forsøget og har fået en kopi af dette samtykkeark, samt en kopi af den skriftlige information om forsøget til eget brug.

Jeg giver samtykke til, at der i forbindelse med forsøget indhentes oplysninger fra min patientjournal samt udveksles oplysninger med mine behandlere i OPUS (behandlende læge, primærbehandler, kontaktperson)

Forsøgspersons navn: ____________________________________________

________________ ____________________________________________

Dato Underskrift

Såfremt der kommer nye, væsentlige helbredsoplysninger frem om dig i forsøget vil du blive informeret. Vil du **frabede** dig information om nye væsentlige helbredsoplysninger, som kommer frem i forsøget, bedes du markere her:________ (sæt x)

**Erklæring fra den, der afgiver information:**

Jeg erklærer, at forsøgspersonen har modtaget mundtlig og skriftlig information om forsøget.

Efter min overbevisning er der givet tilstrækkelig information til, at der kan træffes beslutning om deltagelse i forsøget.

Navn på den, der har afgivet information: ______________________________________

_______________ ____________________________________________

Dato Underskrift
